# The Anatomic Variability of the Lateral Ventricles of the Human Brain Depending on Age and Sex

**DOI:** 10.7759/cureus.45915

**Published:** 2023-09-25

**Authors:** Iuliia Zhuravlova, Anne Montgomery

**Affiliations:** 1 Biomedical Sciences, Mercer University School of Medicine, Macon, USA; 2 Community Medicine, Mercer University School of Medicine, Macon, USA

**Keywords:** sex-related changes, age-related changes, anatomic variability, morphometry, brain computed tomography, lateral ventricles

## Abstract

The evaluation of the different parameters of the brain ventricles is used in the diagnostics of age-related degenerative diseases of the central nervous system, as well as psychiatric disorders. The purpose of this study was to obtain data about the normal morphologic parameters of the lateral ventricles of the human brain and their relations with age and sex to establish a solid background for the diagnostics of any pathologic changes. Computed tomography (CT) studies of 108 healthy individuals aged from 17 to 86 (mean age: 46.87 ± 17.31) were selected for the study. The width of the different portions of the lateral ventricles was measured, and its relations with age and gender were assessed. The study has demonstrated a statistically significant dependence of the width of the different portions of the lateral ventricles on age. There was no statistically significant difference in this parameter identified between male and female groups. The width of the different portions of the lateral ventricles of the brain increases with age. Parameters are similar in males and females.

## Introduction

The ventricles of the human brain are cavities filled with the cerebrospinal fluid. They represent the cavities of the initial embryonic brain vesicles. They are communicating through the system of the foramina and channels, allowing the normal circulation of the cerebrospinal fluid in the system. The lateral ventricles of the brain represent the cavities of the telencephalon and connect with the third ventricle (the cavity of the diencephalon) via the interventricular foramina of Monro [1]. The blockage of these connections between the ventricles and the subarachnoid space, as well as the imbalance between the cerebrospinal fluid production and absorption, can lead to the development of hydrocephalus, which can be communicating or non-communicating, depending on the mechanism of the development [2].

The ventricular system of the human brain is one of the active areas of research nowadays as medical imaging has become widely available around the globe. With the evolvement of computed tomography (CT) and magnetic resonance imaging (MRI), a precise, noninvasive investigation and assessment of the components of the ventricular system have become an accessible and valuable methodology for the diagnostics and research of normal versus abnormal conditions [3]. The evaluation of the different parameters of the ventricles is used in the diagnostics of degenerative diseases of the central nervous system such as Parkinson’s disease [4], Alzheimer’s disease [5], Huntington’s disease [6], and multiple sclerosis [7]. Changes in different parameters of the ventricular system are also a valuable diagnostic factor for certain psychiatric disorders, such as schizophrenia [8], bipolar disorders, and depression [9], as well as general cognitive decline, dementia, and traumatic and malignant lesions [10]. The monitoring of the cerebrospinal fluid pressure and the ventricular parameters may be a helpful tool in adjusting the treatment choices for different conditions. Specifically, controlling the dose of the serotonin reuptake inhibitors in psychiatric patients was studied, and volume reduction was reported after the treatment course [11].

This signifies the importance of understanding the normal anatomic parameters of the ventricular system in order to timely diagnose any pathologic changes in the size, volume, or symmetry of the system [12]. Genetic factors have been reported to have a major role in the heritability of the volume of numerous brain structures, as well as the ventricular volume. The evidence emerged from the studies comparing monozygotic twins and dizygotic twins with and without schizophrenia to healthy controls [13]. The increased width of the temporal horns of the lateral ventricles was reported to be associated with cognitive dysfunction [14].

Numerous studies have concentrated on the investigation of the parameters of the third and fourth ventricles, as well as the lateral ventricles of the brain. The most valuable techniques are those that provide information from patient brain imaging studies [15,16]. There are studies that were conducted on the cadaveric brain, but we think that postmortem changes may suggest certain limitations to the data received from such specimens.

The complex anatomy, deep positioning within the different lobes of the brain hemispheres, and associated choroid plexus vasculature render the surgical treatment of the pathology of the lateral ventricles of the brain a challenging and complicated task. This makes early diagnosis even more desirable as the appropriate interventions preventing further progression of the pathologic changes will become possible. The detailed information about age- and gender-related variability may be of significant value in the process of development of new surgical techniques and approaches to the ventricles, such as ventricular puncture, in order to treat ventricular hemorrhages and other conditions [17].

The purpose of this study is to obtain data about the normal morphologic parameters of the lateral ventricles of the human brain and their relation to age and sex. The potential new and intriguing implementation of the data that we are aiming to investigate lies in the field of space travel. Currently, there are some limited data available showing the significant increase in the volume of the lateral and third ventricles following long-duration spaceflights in astronauts due to microgravity [18], which makes our effort in establishing the normal reference ranges even more valuable in this perspective. The availability of such data will make the process of differentiating normal versus pathologic parameters easier for the related professionals.

## Materials and methods

Initially, we have reviewed a collection of 340 CT imaging studies of the head. This collection was obtained from the radiology department of a private “mother and child” clinic in Luhansk (Ukraine) in 2013. The CT scans were made on the Asteion Super 4 multislice helical CT scanner (image acquisition mode: 2 mm × four rows). The axial slices of the head were scanned parallel to the axis of the lateral ventricle.

We have selected 108 scans of the head. The rest of the imaging studies were excluded based on the following criteria: a past history of congenital intracranial anomalies, psychiatric disorders, dementia and other neurodegenerative disorders, cerebral infarction, trauma, local mass lesions, previous intracranial surgery, and known vascular pathology. This study is based on the analysis of the computed tomography images of healthy subjects aged from 17 to 86 (mean age: 46.87 ± 17.31), including 40 males (37%) and 68 females (63%). Some of the examples of the reasons for which the individuals with normal anatomy of the brain received computed tomography of the head were instances of dizziness that later proved to be unrelated to the disorders of the central nervous systems, episodes of fainting due to metabolic issues, some individuals with Bell’s palsy, persons evaluated for the conditions associated with paranasal sinuses, etc.

**Figure 1:**
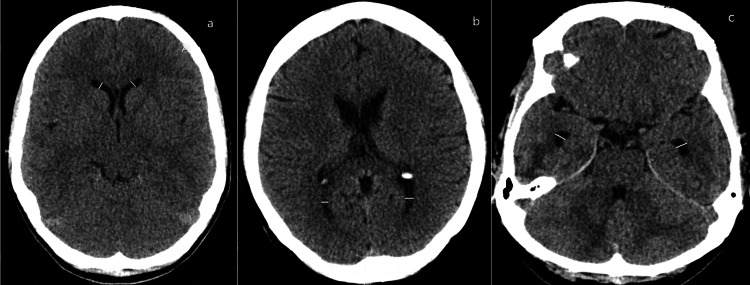
Morphometric measurements of the width of the lateral ventricles. The measurements of the width of the frontal horns of the lateral ventricles in the anterior third (panel a), occipital horns of the lateral ventricles in the posterior third (panel b), and temporal horns of the lateral ventricles in the anterior third (panel c).

**Figure 2:**
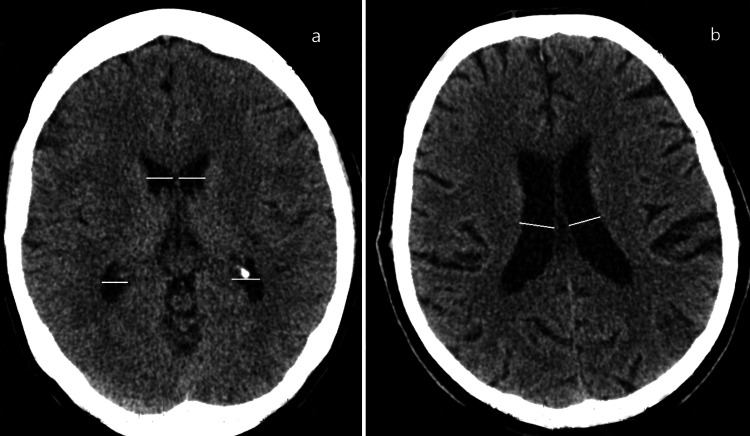
Morphometric measurements of the width of the lateral ventricles. Measurements of the width of the anterior third of the body and antrum of the lateral ventricle (panel a) and the middle third of the body of the lateral ventricle (panel b).

In this study, we focused on the width of the different portions of the lateral ventricles of the brain measured in the axial sections as we have identified that there is a lack of information in the published literature about these parameters with only some limited data available. The width of the anterior horn and temporal horns was measured in their anterior thirds; the width of the occipital horn was measured in the posterior third (Figure 1). The width of the body of the third ventricle was measured in its anterior third and in the middle third, and the width of the atrium was taken (Figure 2). The measurements were taken separately for both the right and left ventricles utilizing the eFilm Lite Merge Healthcare software measurement tools. To minimize the error, the measurements were done by two persons: an experienced radiologist and an expert in neuroanatomy. The inter-rater reliability was assessed with Cohen’s kappa with 92% values being between 0.61-0.80 (good) and 0.81-1.00 (very good) agreement [19]. All patient-identifying information and the specifics regarding the dates and additional information were removed from the images presented in the article to ensure patient data protection.

Statistical tests were conducted using JMP Pro 16.0. Normality tests were performed through Kolmogorov-Smirnov tests; all continuous data were normal (p > 0.05). For continuous data across categories, an independent t-test was used; for categorical data, Pearson’s chi-square test was applied, unless Fischer’s exact test was more appropriate. Simple linear regression analysis was performed for modeling a relationship between the width of the different portions of the lateral ventricles and age. A multivariate analysis of variance (MANOVA) was used to establish a potential sex effect on width differences between the left and right ventricles. Continuous data used Pearson’s R. A point-biserial correlation was used between sex and width variables as well.

The study was performed in compliance with all the ethical standards and approved by the institutional review board (IRB) of the Mercer University as an exempt (protocol number: H2302035).

## Results

In our research, we wanted to assess and present clear and unbiased data about the width of the different portions of the lateral ventricles of the brain and their relation with age and gender. We also wanted to compare the width of the right and left lateral ventricles in the entire sample. The collected data were first explored by analyzing basic statistical parameters. The mean and the standard deviation were assessed in the entire sample, as well as for males and females separately, expressed in centimeters and summarized in Table 1.

**Table 1:** Summary of the width of the different portions of the lateral ventricles of the brain (centimeters). SD: standard deviation

Summary of the width	Entire sample, N = 108 (100%)				Females, N = 68 (63%)				Males, N = 40 (37%)			
	Right		Left		Right		Left		Right		Left	
	Mean	SD	Mean	SD	Mean	SD	Mean	SD	Mean	SD	Mean	SD
Anterior horn	0.41	0.19	0.50	0.22	0.43	0.20	0.51	0.24	0.40	0.17	0.48	0.19
Anterior third of the body	0.74	0.18	0.77	0.24	0.73	0.18	0.76	0.23	0.74	0.19	0.78	0.25
Middle third of the body	1.11	0.31	1.12	0.33	1.12	0.33	1.13	0.34	1.10	0.28	1.10	0.32
Atrium	1.01	0.23	0.99	0.27	1.00	0.24	0.99	0.29	1.01	0.22	0.98	0.24
Occipital horn	0.51	0.16	0.51	0.17	0.51	0.16	0.51	0.18	0.51	0.17	0.52	0.16
Temporal horn	0.66	0.29	0.61	0.27	0.69	0.32	0.63	0.29	0.61	0.21	0.56	0.23

The assessment of the association of the width with the age of the studied individuals, for all the measured portions of the lateral ventricles, has shown that width increases with advancing age (Table 2).

**Table 2:** Correlation of the width of the different portions of the lateral ventricles of the brain with age. Significance levels: ***p < 0.001, **p < 0.01, *p < 0.05, and ^NS^non-significant.

Parameter of the width of a portion of the lateral ventricles	Right		Left	
	Pearson’s R, N = 108 (100%)	p-value	Pearson’s R, N = 108 (100%)	p-value
Anterior horn	0.502	<0.001***	0.549	<0.001***
Anterior third of the body	0.384	<0.001***	0.503	<0.001***
Middle third of the body	0.331	<0.001***	0.488	<0.001***
Atrium	0.277	0.004**	0.401	<0.001***
Occipital horn	0.401	<0.001***	0.451	<0.001***
Temporal horn	0.155	0.113^NS^	0.194	0.046*

**Figure 3:**
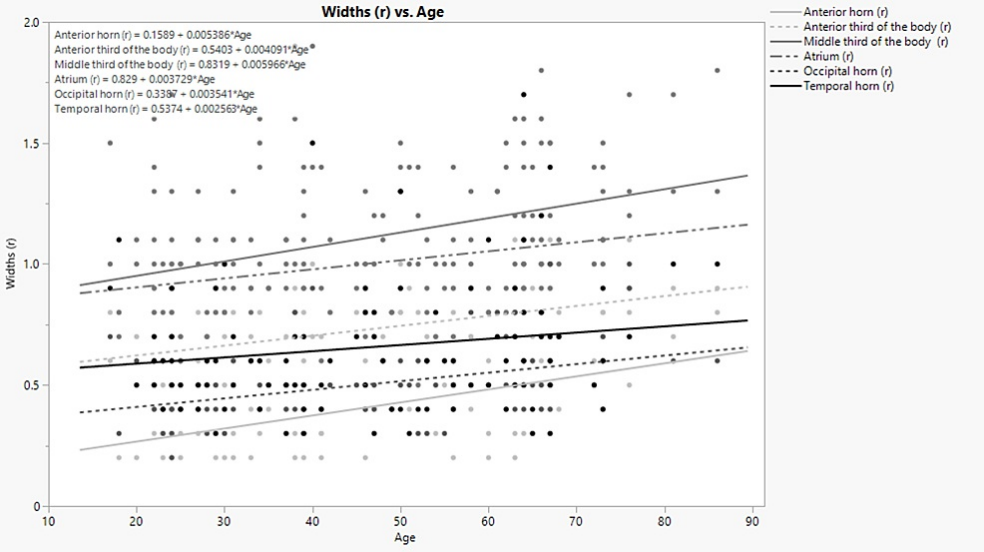
Dependence of the width of the different portions of the right lateral ventricle on age. (r) stands for “right.”

**Figure 4:**
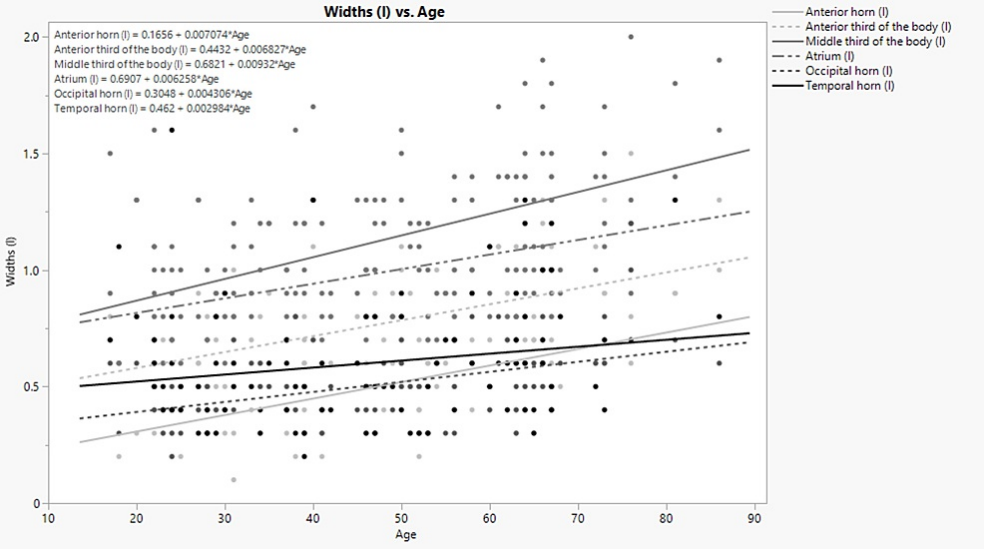
Dependence of the width of the different portions of the left lateral ventricle on age. (l) stands for “left.”

The width of the anterior horns and occipital horns of the right and left ventricles has moderate positive correlation with age. The width of the anterior third, the middle third of the body of the left ventricle, and the antrum of the left ventricle has also shown to have moderate positive correlation with age. The width of the anterior and middle thirds of the right ventricle and the antrum of the right ventricle and the width of the temporal horns of both the right and left ventricles have shown to have a weak positive correlation with age. The data were statistically significant except for the correlation of the width of the temporal horn of the right ventricle with age. The simple linear regression analysis has shown how the width of the different portions of the right and left ventricles can be predicted by age. The graphic representation and corresponding formulas for each portion are shown on the Figure 3 and Figure 4.

When we have analyzed the difference between the width parameters of the different portions of the right and left ventricle utilizing dependent t-test, only the frontal horns, anterior third of the body, and temporal horns of the right and left ventricles have demonstrated statistically significant differences with the prevalence of the width of the right frontal horn and the width of the anterior third of the body of the right ventricle over the left ventricle. The temporal horn of the left ventricle was found to be wider than the temporal horn of the right ventricle. The difference between the width of the middle third of the body, atrium, and occipital horns of the right and left ventricles was found to be statistically non-significant (Table 3).

**Table 3:** Comparison of the parameter of the width of the different portions of the right and left lateral ventricles (centimeters). Significance levels: ***p < 0.001, *p < 0.05, and ^NS^non-significant. SD: standard deviation

Portion of the lateral ventricle	Width mean (SD), right	Width mean (SD), left	p-value
Anterior horn	0.50 (0.22)	0.41 (0.19)	<0.001***
Anterior third of the body	0.77 (0.24)	0.74 (0.18)	0.025*
Middle third of the body	1.12 (0.33)	1.11 (0.31)	0.546^NS^
Atrium	0.99 (0.27)	1.01 (0.23)	0.377^NS^
Occipital horn	0.51 (0.17)	0.51 (0.16)	0.734^NS^
Temporal horn	0.61 (0.27)	0.66 (0.29)	<0.001***

As the next step, we have analyzed how the parameter of the width of the different portions of the lateral ventricle depends on sex. The MANOVA has demonstrated that no statistically significant differences exist between males and females compared to the entire sample, meaning that males and females follow a similar trend as the entire sample (Table 4).

**Table 4:** MANOVA for sex effect on the widths of the different portions of the right and left lateral ventricles (centimeters). SD, standard deviation; NS, non-significant; MANOVA, multivariate analysis of variance

Portion of the lateral ventricle	Width mean (SD), right		Width mean (SD), left		p-value
	Female	Male	Female	Male	
Anterior horn	0.43 (0.20)	0.40 (0.17)	0.51 (0.24)	0.48 (0.19)	0.997^NS^
Anterior third of the body	0.73 (0.18)	0.74 (0.19)	0.76 (0.23)	0.78 (0.25)	0.842^NS^
Middle third of the body	1.12 (0.33)	1.10 (0.28)	1.13 (0.34)	1.10 (0.32)	0.642^NS^
Atrium	1.00 (0.24)	1.01 (0.22)	0.99 (0.29)	0.98 (0.24)	0.594^NS^
Occipital horn	0.51 (0.16)	0.51 (0.17)	0.51 (0.18)	0.52 (0.16)	0.977^NS^
Temporal horn	0.69 (0.32)	0.61 (0.21)	0.63 (0.29)	0.56 (0.23)	0.421^NS^

We also tried to utilize a different approach to investigate the relations between the widths and sex with looking at point-biserial correlation. This method has also demonstrated that no statistically significant relations exist between these variables (Table 5).

**Table 5:** Point-biserial correlation between sex and width variables. NS: non-significant

Portion of the lateral ventricle	Right		Left	
	Point-biserial correlation	p-value	Point-biserial correlation	p-value
Anterior horn	-0.071	0.465^NS^	-0.057	0.556^NS^
Anterior third of the body	0.010	0.921^NS^	-0.169	0.828^NS^
Middle third of the body	-0.022	0.823^NS^	-0.044	0.650^NS^
Atrium	0.006	0.947^NS^	-0.021	0.831^NS^
Occipital horn	0.007	0.945^NS^	0.005	0.959^NS^
Temporal horn	-0.152	0.117^NS^	-0.124	0.202^NS^

## Discussion

Reviewing current literature related to the morphologic analysis of the lateral ventricles of the brain, we have found numerous studies implementing different techniques, as well as investigating different portions and parameters of the ventricular system.

Multiple studies focused on the length of the different portions of the lateral ventricles of the brain utilizing computed tomography [20-23], as well as the dissection method [24]. In our understanding, the complex spatial orientation of the lateral ventricles with the curves of the frontal and temporal horns, as well as the body of the ventricle laterally from the sagittal plane and downward from the horizontal plane, does not allow to receive valid longitudinal measurements of the portions of the lateral ventricles in the axial sections without the three-dimensional (3D) reconstruction or the use of the cadaveric material where the length is directly measured on the brain specimens. This was the reason for us to focus on the transverse parameter, the width, which is easily measured in the axial sections. In the study by Agegnehu et al. [25], the length and width of the frontal horn were assessed in a Northwest Ethiopia population. The authors reported the width of the right frontal horn to be 5.04 ± 1.56 mm in males and 4.51 ± 1.45 mm in females and of the left frontal horn to be 5.22 ± 1.61 mm in males and 4.68 ± 1.42 in females. Their findings were statistically significant with a p-value of <0.05. In our study, the difference between the widths of the frontal horns in males and females did not reach statistical significance.

In the study by Polat et al. [12], a different technique was utilized. The authors measured the combined width of the right and left frontal horns and reported the following data: the mean of the frontal horn width was 33.14 ± 3.06 in females and 34.85 in females. The reported difference was statistically significant with a p-value of <0.001. In our study, we aimed to look separately at the right and left lateral ventricle portions and to compare the widths of the right and left sides.

In several studies, a different approach to analyzing lateral ventricles was utilized. The authors have implemented different ratio assessments, specifically the frontal horn ratio, the bicaudate ratio, and Evan’s ratio [26-28]. We abstained from using the frontal horn ratio in our analysis as we believe that it may be influenced by the shape and curve of the frontal horn between the anatomic planes of reference (sagittal, frontal, and horizontal). We focused specifically on the actual widths of each horn of the lateral ventricle, as well as the three different portions of the body of the lateral ventricle. We believe that it gives a clearer idea of these ventricular parameters without being influenced by their relations with surrounding structures. The use of the bicaudate ratio in our view gives a limited understanding of the ventricular width and may be influenced by the presence of the cavum septi. The bicaudate ratio is more representative of caudate nuclei volume rather than ventricular volume. We have also decided not to analyze Evan’s ratio as it does not allow us to see the difference between the right and left lateral ventricles, only gives a general idea about the ventricular enlargement for both ventricles at once, and may be affected by the different imaging planes and angles [29].

We suggest that the data we obtained in our study may be useful for understanding the normal anatomic parameters of the ventricular system, which is a must for physicians and scientists to timely diagnose and best treat any pathologic changes in the size, volume, or symmetry of the system. The detailed evaluation of the brain ventricular parameters could be very helpful in this era of developing long-distance robotic surgery [30]. Potentially, putting this information from our research and from other similar types of research into a program, analyzing it by software, and using it for correcting the position of the robotic equipment may prove extremely valuable in the future.

## Conclusions

We have identified that statistically significant changes in the width of the different portions of the lateral ventricles of the brain occur in association with age. The width of the frontal horns, occipital horns, temporal horns, and the anterior and middle third of the body and antrum of the lateral ventricles increases with advancing age. The data were statistically significant for all the measured portions of the lateral ventricles except for the temporal horn of the right ventricle.

Significant differences were found between the widths of the frontal and temporal horns and the anterior third of the body of the right and left ventricles, with frontal horns and the anterior third of the body being wider on the right side and the temporal horn width being wider on the left side. No statistically significant differences were found in the width of the different portions of the right and left lateral ventricles of the brain when comparing female to male samples.
